# Influenza A–Associated Thrombotic Microangiopathy With Normal a Disintegrin and Metalloproteinase With Thrombospondin Type 1 Motif 13 (ADAMTS13) in a Young Male Patient: A Case Report

**DOI:** 10.7759/cureus.110428

**Published:** 2026-06-08

**Authors:** Amna Sayah Abdulla Alhammadi, Noura Nasir Awasia Alzaabi, Emadullah Raidullah, Jonathan Price

**Affiliations:** 1 Internal Medicine, Sheikh Shakhbout Medical City, Abu Dhabi, ARE; 2 Hematology, Sheikh Shakhbout Medical City, Abu Dhabi, ARE

**Keywords:** acquired ttp, adamts13, influenza a, plasma exchange therapy, rapid influenza, seasonal influenza, secondary tma, thrombotic microangiopathy, thrombotic microangiopathy (tma), ttp (thrombotic thrombocytopenic purpura)

## Abstract

Thrombotic microangiopathy (TMA) is a life-threatening syndrome characterized by microangiopathic hemolytic anemia, thrombocytopenia, and end-organ injury resulting from widespread microvascular thrombosis. It encompasses several entities, including thrombotic thrombocytopenic purpura (TTP), atypical hemolytic uremic syndrome (aHUS), and secondary forms triggered by infections, medications, or systemic disease. Viral infections, including influenza A, have been increasingly recognized as rare triggers of secondary TMA, through endothelial injury and immune dysregulation, even in the absence of severe a disintegrin and metalloproteinase with thrombospondin type 1 motif 13 (ADAMTS13) deficiency. We report a case of a 21-year-old male patient who presented with fever, productive cough, and cola-colored urine following a positive influenza A antigen test. He was found to have severe thrombocytopenia, microangiopathic hemolytic anemia with schistocytes on peripheral smear, markedly elevated lactate dehydrogenase, acute kidney injury, and concurrent rhabdomyolysis. ADAMTS13 activity was 0.8 IU/mL (equivalent to 80% activity), within the normal range, and consistent with secondary influenza A-triggered TMA. The patient was treated with therapeutic plasma exchange and high-dose intravenous methylprednisolone, achieving full hematological recovery. This case highlights the importance of recognizing influenza A as a trigger of secondary TMA with a TTP-like phenotype despite normal ADAMTS13 activity, and underscores the critical role of early empiric plasma exchange as a life-saving intervention.

## Introduction

Thrombotic microangiopathy (TMA) is a life-threatening syndrome characterized by microangiopathic hemolytic anemia (MAHA), thrombocytopenia, and end-organ ischemia resulting from widespread microvascular thrombosis [1]. The major subtypes include thrombotic thrombocytopenic purpura (TTP), driven by severe a disintegrin and metalloproteinase with thrombospondin type 1 motif 13 (ADAMTS13) deficiency; atypical hemolytic uremic syndrome (aHUS), mediated by complement dysregulation; and secondary TMA, which arises in the context of infections, medications, malignancy, pregnancy, or autoimmune disease [2]. Each subtype shares a common pathological endpoint of microvascular platelet-rich thrombi, yet differs substantially in underlying mechanisms, diagnostic criteria, and therapeutic approaches.

Secondary TMA triggered by viral infections represents a diagnostically challenging and potentially underrecognized entity. Influenza A virus has been increasingly reported as a rare trigger of TMA, inducing endothelial injury, cytokine-driven platelet activation, and immune dysregulation that collectively produce a TTP-like clinical phenotype, even in the absence of severe ADAMTS13 deficiency [3,4]. This presents a significant diagnostic challenge, as normal ADAMTS13 activity may lead clinicians away from initiating empiric plasma exchange, despite the clinical urgency of the situation.

We report a case of secondary TMA triggered by an influenza A infection in a previously healthy young male patient, presenting with a TTP-like phenotype, concurrent rhabdomyolysis, and normal ADAMTS13 activity, who achieved full hematological recovery following therapeutic plasma exchange and high-dose corticosteroids.

## Case presentation

A 21-year-old male patient with no known past medical history developed upper respiratory symptoms and visited a private clinic four days after symptom onset with a productive cough, rhinorrhea, sore throat, nausea, vomiting, and high-grade fever of 39°C. A point-of-care rapid antigen test was positive for influenza A, and he was prescribed oseltamivir 75 mg twice daily. He returned to the emergency department the following day due to persistent symptoms and dark cola-colored urine that he had noticed for several days. He had visited the emergency department two days prior and received intramuscular ketorolac for pain, with a repeat dose administered on this visit.

On admission, the patient was febrile (38.2°C; reference range: 36.1-37.2°C) and tachycardic at 106 beats per minute (reference range: 60-100 bpm). Blood pressure was 117/60 mmHg (reference range: 90-120/60-80 mmHg) with a mean arterial pressure of 79 mmHg (reference value: ≥65 mmHg). Respiratory rate was 18 breaths per minute (reference range: 12-20 breaths/min), and oxygen saturation was 97% on room air (reference value: ≥95% on room air). He was alert and oriented with no focal neurological deficits. There was no jaundice, rash, or lymphadenopathy. Cardiorespiratory and abdominal examinations were unremarkable.

Laboratory investigations on admission are detailed in Table 1.

**Table 1:** Blood investigations on admission eGFR: estimated glomerular filtration rate; CKD-EPI: Chronic Kidney Disease Epidemiology Collaboration; LDH: lactate dehydrogenase; INR: international normalized ratio; ADAMTS13: a disintegrin and metalloproteinase with thrombospondin type 1 motif 13; APTT: activated partial thromboplastin time; PT: prothrombin time. Arrows indicate deviation from normal (↑ elevated, ↓ reduced, ↑↑/↓↓ critically abnormal).

Parameter	Normal range	Value at admission
Hemoglobin (g/L)	130-170	118 ↓
Platelets (x10⁹/L)	150-400	19 ↓↓
White blood cells (x10⁹/L)	4.0-11.0	4.81
Serum creatinine (µmol/L)	62-106	215 ↑
eGFR - CKD-EPI (mL/min/1.73m²)	>60	52 ↓
Sodium (mmol/L)	136-145	144
Bilirubin total (µmol/L)	3-21	32.3 ↑
Bilirubin direct (µmol/L)	0-5	5.4 ↑
LDH (U/L)	135-225	2808 ↑↑
INR	0.9-1.1	2.38 ↑
APTT (seconds)	25-40	41.9 ↑
PT (seconds)	10-14	29.50 ↑
Fibrinogen (g/L)	2.0-4.0	0.40 ↓↓
ADAMTS13 activity (IU/mL)	0.7-1.2	0.8 (Normal)

Peripheral blood smear demonstrated two+ schistocytes, confirming microangiopathic hemolysis. Urine myoglobin was 169 μg/L with heavy granular casts (41 per high-power field), indicating concurrent rhabdomyolysis and acute tubular necrosis. ADAMTS13 activity was 0.8 IU/mL, within the normal range. Influenza A antigen by point-of-care testing was positive. 

Chest X-ray showed no pulmonary consolidation, pleural effusion, or pneumothorax. Abdominal ultrasound demonstrated mild hepatic steatosis with no other significant findings.

Immunological and serological investigations are shown in Table 2.

**Table 2:** Immunology and serology investigations ANA: antinuclear antibody; c ANCA: cytoplasmic anti-neutrophil cytoplasmic antibody; PR3: proteinase-3; p ANCA: perinuclear anti-neutrophil cytoplasmic antibody; MPO: myeloperoxidase; C3/C4: complement components; ASO: anti-streptolysin O; HIV: human immunodeficiency virus. Arrows indicate deviation from normal (↑ elevated, ↓ reduced).

Parameter	Normal range	Value
ANA	<5	4 (Negative)
c ANCA - PR3 (IU/mL)	<5	<2 (Negative)
p ANCA - MPO (IU/mL)	<5	<3 (Negative)
C3 (g/L)	0.90-1.80	1.59
C4 (g/L)	0.16-0.38	0.19
C-reactive protein (mg/L)	<5	3.20
Rheumatoid factor (IU/mL)	<14	<10 (Negative)
ASO quantitative (IU/mL)	<200	<50 (Negative)
HIV Ag/Ab screen	Negative	Negative
Hepatitis B surface antigen	Negative	Negative
Hepatitis B core antibody	Negative	Negative
Hepatitis C antibody screen	Negative	Negative
Complement total (U/mL)	30–75	58 (Normal)
Complement C3 (mg/dL)	75–175	220 ↑
Complement C4 (mg/dL)	14–40	33 (Normal)
Factor B complement antigen (mg/dL)	15.2–42.3	>58.8 ↑
Factor H complement antigen (mg/dL)	18.5–40.8	53.1 ↑
CBb complement (mcg/mL)	<1.7	>6.0 ↑↑
SC5b-9 (ng/mL)	<251	587 ↑↑
Alternative complement pathway function (%)	≤46	88 ↑

Autoimmune and viral hepatitis screens were negative. Complement studies revealed elevated sC5b-9 and factor Bb, consistent with secondary alternative pathway activation; however, the expected findings of primary aHUS (low factor H, normal C4, and low alternative pathway hemolytic 50 (AH50)) were not present. 

The PLASMIC score was calculated as five out of seven, reflecting intermediate probability of severe ADAMTS13 deficiency, as detailed in Table 3. 

**Table 3:** PLASMIC score calculation A PLASMIC score of 5 corresponds to intermediate probability of severe ADAMTS13 deficiency (approximately 5-25%). Despite the intermediate score, therapeutic plasma exchange was initiated empirically given the clinical severity, consistent with current guidelines recommending prompt treatment in clinically suspected TTP regardless of score. MCV: mean corpuscular volume; INR: international normalized ratio.

Criterion	Patient's value	Points
Platelet count <30 x 10⁹/L	19 x 10⁹/L	1
Hemolysis (elevated bilirubin, undetectable haptoglobin, or reticulocytosis)	Bilirubin 32.3 µmol/L	1
No active cancer	None	1
No solid organ or stem cell transplant	None	1
MCV <90 fL	82.3 fL	1
INR <1.5	2.38	0
Creatinine <2.0 mg/dL (177 µmol/L)	215 µmol/L	0
Total PLASMIC Score		5 / 7

Upon establishing the diagnosis of TMA, therapeutic plasma exchange was initiated urgently using 5% human albumin as replacement fluid. Fresh frozen plasma was intentionally avoided given the normal ADAMTS13 activity, as albumin was preferred to remove pathogenic antibodies and complement mediators without replenishing coagulation factors. Each session targeted one to 1.5 plasma volumes, performed via central venous access with acid citrate dextrose, formula A (ACD-A) anticoagulation. Intravenous methylprednisolone 1,000 mg was administered concurrently as adjunct immunosuppression for three days, targeting removal of circulating antibodies and complement factors contributing to endothelial activation and platelet consumption. Fibrinogen concentrate was administered prior to each session given the critically low fibrinogen of 0.40 g/L. Plasma exchange was continued daily until platelet count exceeded 150 x 10⁹/L for two to three consecutive days, lactate dehydrogenase (LDH) approached normal, and clinical improvement was sustained.

Following three sessions of plasma exchange and three days of high-dose corticosteroids, the patient demonstrated dramatic clinical and laboratory recovery, as shown in Table 4.

**Table 4:** Laboratory findings following three plasma exchange sessions Values represent the best post-exchange laboratory results obtained on November 1-2, 2025, following three daily therapeutic plasma exchange sessions and three days of intravenous methylprednisolone 1,000 mg. eGFR: estimated glomerular filtration rate; CKD-EPI: Chronic Kidney Disease Epidemiology Collaboration; LDH: lactate dehydrogenase; CK: creatine kinase; AST: aspartate aminotransferase; ALT: alanine aminotransferase; INR: international normalized ratio; APTT: activated partial thromboplastin time; PT: prothrombin time. Arrows indicate deviation from normal (↑ elevated, ↓ reduced).

Parameter	Normal range	Post-exchange value
Hemoglobin (g/L)	130-170	95 ↓
Platelets (x10⁹/L)	150-400	341 (normalized)
White blood cells (x10⁹/L)	4.0-11.0	7.78
Serum creatinine (µmol/L)	62-106	139 ↑ (improving)
eGFR - CKD-EPI (mL/min/1.73m²)	>60	62 (improving)
Sodium (mmol/L)	136-145	141
Potassium (mmol/L)	3.5-5.1	4.51
Chloride (mmol/L)	98-107	112 ↑
CO2 (mmol/L)	22-29	22
Bilirubin total (µmol/L)	3-21	11.2 (improving)
Bilirubin direct (µmol/L)	0-5	6.4 ↑
Total protein (g/L)	64-83	54 ↓
Albumin (g/L)	35-50	46
Total globulin (g/L)	20-35	8.0 ↓
Alkaline phosphatase (IU/L)	40-150	16 ↓
AST (IU/L)	10-40	14
ALT (IU/L)	7-56	12
LDH (U/L)	135-225	273 ↑ (improving)
Total CK (IU/L)	30-200	189
INR	0.9-1.1	1.18 (improving)
APTT (seconds)	25-40	30.9 (improving)
PT (seconds)	10-14	15.60 ↑ (improving)
Fibrinogen (g/L)	2.0-4.0	4.14 (normalized)
ADAMTS13 activity (IU/mL)	0.7-1.2	Not repeated

**Figure 1:**
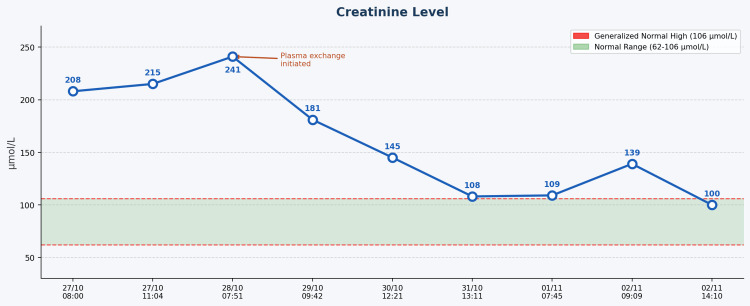
Serial serum creatinine levels during the hospital stay Serial serum creatinine measurements from admission through discharge demonstrating peak at 241 µmol/L on day two of admission followed by progressive improvement to 100 µmol/L approaching near-normal range by discharge. Red dashed lines indicate the generalized normal reference range (62–106 µmol/L). The arrow marks initiation of therapeutic plasma exchange.

Serial creatinine levels throughout the admission are detailed in Figure 1 , demonstrating progressive renal recovery from a peak of 241 µmol/L to near-normal levels by discharge.

The patient became afebrile and hemodynamically stable, reporting marked subjective improvement with no new complaints by day five of hospitalization.

## Discussion

TMA is a syndrome characterized by the triad of thrombocytopenia, microangiopathic hemolytic anemia, and organ damage resulting from platelet-rich thrombi in the microvasculature. It encompasses primary forms such as TTP and aHUS, as well as secondary TMA arising from infections, medications, autoimmune disease, and malignancy [1,2]. Pathologically, it involves endothelial injury and microvascular thrombosis, the trigger and mechanism of which determine the diagnostic classification and therapeutic approach. In this case, the clinical presentation, temporal relationship with confirmed Influenza A infection, and dramatic response to plasma exchange were collectively consistent with secondary TMA.

The ADAMTS13 activity of 0.8 IU/mL (80% activity) is a key finding in this case. While preserved ADAMTS13 activity argues against classic autoimmune TTP, it does not exclude a TTP-like presentation within the broader TMA spectrum. In our patient, the most plausible cause, given the temporal relationship and confirmed positive antigen test, was influenza A infection. Influenza A has been reported to trigger TMA by inducing endothelial injury, cytokine storm-mediated platelet activation, and direct impairment of ADAMTS13 function without the production of inhibitory IgG autoantibodies [4-6]. This is supported by Kubo et al., who reported a similar case of influenza A-triggered TMA without severe ADAMTS13 deficiency that responded successfully to plasma exchange [5]. The presentation in our patient is therefore best classified as secondary TMA with a TTP-like phenotype triggered by the influenza A infection.

This case highlights that a normal ADAMTS13 activity must not deter the clinician from diagnosing and treating TMA when the clinical picture is compelling. Delayed initiation of plasma exchange is the primary determinant of mortality in TMA, and empiric treatment must not await confirmatory laboratory results when clinical suspicion is high [7]. The PLASMIC score of five out of seven in this patient reflected an intermediate probability of severe ADAMTS13 deficiency; however, given the clinical severity and organ involvement, plasma exchange was initiated empirically without delay [8]. As the development of secondary TMA can be attributed to various causes and mechanisms, further investigation into the efficacy of plasma exchange for each specific underlying cause, including viral triggers, is needed to guide clinical decision-making in these complex presentations.

## Conclusions

Influenza A infection can trigger secondary thrombotic microangiopathy with a TTP-like phenotype, presenting with severe thrombocytopenia, microangiopathic hemolytic anemia, and acute organ injury despite normal ADAMTS13 activity. Secondary TMA remains a hematological emergency in which early recognition and prompt initiation of therapeutic plasma exchange combined with high-dose glucocorticoids may be life-saving when the clinical picture is highly suggestive of TTP. Clinicians should maintain a high index of suspicion for secondary TMA in patients presenting with the characteristic triad following viral illness and should not delay treatment while awaiting confirmatory laboratory results. The rapid hematologic recovery observed in this case highlights the importance of timely empiric therapy in virus-triggered TMA.
